# Recovery of neurophysiological measures in post-COVID fatigue: a 12-month longitudinal follow-up study

**DOI:** 10.1038/s41598-024-59232-y

**Published:** 2024-04-17

**Authors:** Natalie J. Maffitt, Maria Germann, Anne M. E. Baker, Mark R. Baker, Stuart N. Baker, Demetris S. Soteropoulos

**Affiliations:** Faculty of Medical Sciences, Newcastle University, Newcastle upon Tyne, NE2 4HH UK

**Keywords:** Motor control, Neurophysiology, Neural circuits

## Abstract

One of the major consequences of the COVID-19 pandemic has been the significant incidence of persistent fatigue following resolution of an acute infection (i.e. post-COVID fatigue). We have shown previously that, in comparison to healthy controls, those suffering from post-COVID fatigue exhibit changes in muscle physiology, cortical circuitry, and autonomic function. Whether these changes preceded infection, potentially predisposing people to developing post-COVID fatigue, or whether the changes were a consequence of infection was unclear. Here we present results of a 12-month longitudinal study of 18 participants from the same cohort of post-COVID fatigue sufferers to investigate these correlates of fatigue over time. We report improvements in self-perception of the impact of fatigue via questionnaires, as well as significant improvements in objective measures of peripheral muscle fatigue and autonomic function, bringing them closer to healthy controls. Additionally, we found reductions in muscle twitch tension rise times, becoming faster than controls, suggesting that the improvement in muscle fatigability might be due to a process of adaptation rather than simply a return to baseline function.

## Introduction

In May 2023, more than three years into the pandemic, the World Health Organization (WHO) Emergency Committee on COVID-19 recommended that the pandemic no longer represented a public health emergency of international concern^1^. However, while acute COVID-19 may no longer constitute a global emergency, the long-term effects of severe acute respiratory syndrome coronavirus 2 (SARS-CoV-2) infection are now becoming apparent. Although the majority of infected individuals experience mild symptoms or recover within a few weeks, a significant proportion continue to experience persistent symptoms beyond the acute phase^2–4^, a condition commonly known as long COVID or post-acute sequelae of SARS-CoV-2 infection.

Long COVID is characterized by a diverse range of symptoms, including fatigue, cognitive impairment, respiratory issues, and musculoskeletal problems^5–7^. Fatigue in long COVID is a complex and multi-faceted symptom, including difficulties in concentrating (‘brain fog’) and a persistent feeling of tiredness, but physical weakness and a reduced ability to exercise is also a key component. Muscle pathology has emerged as a common and debilitating feature, even in individuals with a history of mild COVID-19^8–10^. Reports highlight muscle weakness, myalgia, and impaired motor coordination among long COVID patients^2,5,11^, significantly impacting their quality of life and functional abilities.

To form a comprehensive understanding of the mechanisms underlying long COVID, longitudinal studies are crucial. These allow the evaluation of clinical, immunological, radiological and neurophysiological changes over time, providing insights into the patterns of recovery of these metrics. Comparison of recovery time courses between clinical measures and biomarkers could potentially address whether abnormal measures play a causal role in the disease, or are simply correlated with its downstream consequences.

In a previous study^12^ in long COVID after non-severe SARS-CoV-2 infection, where there had been no requirement for acute hospital in-patient care, we demonstrated changes in muscle physiology. In addition, we found reduced responsiveness in some of the neural circuitry governing cortical excitability, and a rise in the resting heart rate probably indicating an altered balance between sympathetic and parasympathetic systems. These changes might explain some of the symptoms of fatigue described by patients. Here we report a longitudinal follow-up study in a subset of the same cohort, in whom neurophysiological measurements and the self-reported perception of the impact of fatigue on daily life were repeated twice more, at 6 month intervals. The reported impact of fatigue improved significantly after a year; for most of the neurophysiological metrics there was likewise a return to levels seen in age and sex-matched controls. We therefore suggest that these changes in muscle physiology and autonomic function occur as a consequence of a SARS-CoV-2 infection, rather than a pre-existing phenotype that increased susceptibility to developing post-COVID fatigue.

## Methods

Our previous publication^12^ employed 35 non-invasive behavioural and neurophysiological tests to assess specific circuits within the central, peripheral and autonomic nervous systems. In this longitudinal follow up study, we used only the sub-set of these tests which were significantly different between people with pCF and controls in our original paper. Transcranial Magnetic Stimulation (TMS) allowed us to probe the state of cortical motor circuits. Electrical stimulation of muscles assessed peripheral fatigue, recordings of heart rate assessed the state of the autonomic nervous system, and blood oxygen saturation (SaO_2_) was also collected. Participants completed a fatigue impact scale (FIS) questionnaire via a web-based survey tool.

### Participants

The study was approved by the Ethics Committee of Newcastle University Faculty of Medical Sciences; participants provided written informed consent. The study was performed in accordance with the guidelines established in the Declaration of Helsinki, except that the study was not preregistered in a database.

Measures collected in our initial study^12^ were used here as baseline data. This included data from a cohort of 37 participants (27 female) who were suffering from pCF by self-report and a second cohort of 52 volunteer controls (37 female) with no symptoms of fatigue. Inclusion criteria were age 18–65 years, with no history of neurological disease. The first visit to the laboratory was made 6–26 weeks after infection for the pCF cohort. In the control cohort, six subjects had reported having mild COVID-19 but with complete recovery and no symptoms of pCF. Of the 37 people with pCF from our initial study, 18 were later recruited to this longitudinal follow-up study (13 female), completing a further two lab visits at intervals of approximately 6 months to yield a total of three visits.

### General electrophysiological methods

Electromyographic activity (EMG) was recorded with adhesive surface electrodes positioned over muscles (Kendall H59P, Covidien, Dublin, Ireland) using an isolated amplifier (D360, Digitimer, Welwyn Garden City, UK; gain 500, bandpass 30 Hz–2 kHz). Transcranial magnetic brain stimulation (TMS) was given with a Bistim 200^2^ stimulator and figure-of-eight coil (7 cm diameter for each winding; Magstim Company Limited, Whitland, UK), with the coil held tangential to the head at around 45° to the parasagittal plane, inducing current in the brain from posterior to anterior. Coil position relative to the head was maintained using a Brainsight neuronavigation system (Brainbox, Cardiff, UK). Stimulus timing was controlled by a Power1401 intelligent laboratory interface running Spike2 software (Cambridge Electronic Design, Cambridge, UK), which also sampled EMG, force (filter setting DC- 30 Hz, sensitivity 1.5 mV/N) and markers indicating auditory cue onset to hard disk (sampling rate 5 kSamples/s). All measurements were made on the self-reported dominant side. Offline analysis was performed with custom scripts written in the MATLAB programming environment.

### Paired-pulse TMS

EMG was recorded from the first-dorsal interosseous (1DI); the TMS coil was moved to locate the hot spot for this muscle. The resting motor threshold (RMT) was determined, as the intensity required to generate MEPs of amplitude greater than 100 μV on 3/6 sweeps. The test stimulus intensity was set to generate MEP amplitudes of 1 mV, or to 1.2xRMT^13^, whichever was lower, to avoid ceiling effects. The conditioning stimulus intensity was 0.8xRMT. We then measured the responses to the test stimulus alone, and when preceded by the conditioning stimulus at intervals of 10 ms, corresponding to intracortical facilitation (ICF) (see Baker et al., 2023 Supplementary Fig. 1). Twenty repetitions of each condition were given, in pseudo-random order, with the subject at rest. Offline analysis measured the peak-to-peak amplitude of responses to conditioned stimuli as a percentage of the responses to test stimulus alone, yielding the measure *TMS_ICF*.

### Heart rate

A single channel electrocardiogram (ECG) recording was made, using a differential recording from either left shoulder and right leg, or left and right shoulders (bandpass 0.3–30 Hz, gain 500, sampling rate 1 kSamples/s). The ECG was processed offline to extract the time of each QRS complex and compute the mean heart rate (measure *Mean_HR*). Heart rate measures were made during a Stop Signal Reaction Time test, which ensured that the subject was sitting quietly, while engaged in a consistent behaviour.

### Measures of muscle physiology

Our original study^15^ used the twitch interpolation procedure, which allows assessment of an individual’s ability to activate a muscle maximally voluntarily, before and after a sustained (fatiguing) contraction^14^. The experimental sequence followed previous work from this laboratory^15^. Of the multiple measures which that protocol yielded, only peripheral fatigue was found to be significantly different between pCF and controls. Although peripheral fatigue alone could be measured with a simpler procedure, in the follow-up visits reported here we still followed the original full twitch interpolation protocol to ensure that measurements would be entirely comparable between the first and subsequent visits. Subjects sat with their dominant arm and forearm strapped into a dynamometer to measure torque about the elbow; the shoulder was flexed, and the elbow at a right angle, so that the upper arm was horizontal and the forearm vertical. The forearm was supinated. Thin stainless-steel plate electrodes (size 30 × 15 mm) were wrapped in saline-soaked cotton gauze and taped over the belly of the biceps muscle (cathode) and its distal tendon (anode). Electrical stimuli were delivered through these electrodes while monitoring the evoked twitch response recorded by the dynamometer, and the intensity increased until the response grew no further. This supramaximal stimulus was used for all subsequent measurements.

The following recordings were then made in sequence; this protocol was followed to maintain consistency with our original study. A brief tone cued the subject to produce and hold a maximal voluntary contraction; 2 s after the tone, a stimulus was given to the biceps, and 1 s later a second tone indicated that the subject should relax. Five seconds later, a biceps stimulus was given, followed by a further 55 s rest period. This sequence was repeated three times. A long tone then cued the subject to make a sustained maximal voluntary contraction. This was continued either for 95 s, or until the force exerted fell below 60% of the initial maximal level. During this sustained contraction, the biceps was stimulated every 10 s. After the contraction ended, a final three biceps stimuli were given at rest (inter-stimulus interval 5 s).

From the three stimuli delivered at rest at the start, we averaged the maximal twitch at rest and measured its amplitude, ${F}_{rest}^{before}$. From the three stimuli delivered at rest after the sustained contraction, we measured ${F}_{rest}^{after}$.

Peripheral fatigue (measure *TI_PeriphFatigue*) was calculated as:

$$TI\text{\_}PeriphFatigue=\frac{{F}_{rest}^{after}}{{F}_{rest}^{before}} 100\%$$

This describes the reduced ability of the muscle to generate force after fatigue, even when activation is performed independent of the central nervous system by an electrical stimulus to the muscle. Additionally, we measured the time to maximal force generation following direct electrical stimulation to the muscle (measure *RiseTime*) using the twitch evoked at rest at the start of the procedure, before the fatiguing contraction.

### Biometric data

Blood oxygen saturation was measured using a pulse oximeter placed onto the index finger (*SaO*_*2*_). This was recorded just after the TMS measurements, when the subject had been sitting at rest for around 20 min, and before the measure of peripheral fatigue.

### Statistics

Descriptive statistics are given as mean ± standard deviation (SD). Normality was tested using Kolmogorov–Smirnov tests. For parametric data, comparisons for each metric across visits were carried out using a repeated measures ANOVA (using the ‘fitrm’ function in MATLAB). For nonparametric data, a Friedman test was used instead. Correlation between measures was assessed with linear regression and results reported as r^2^ values. Paired t-tests, or nonparametric Wilcoxon sign-rank tests, were used to compare individual measures between visits. Unpaired t-tests, or nonparametric Wilcoxon rank-sum tests, were used to compare measures between different cohorts. The Benjamini–Hochberg procedure was used to correct for multiple comparisons.

## Results

**Figure 1:**
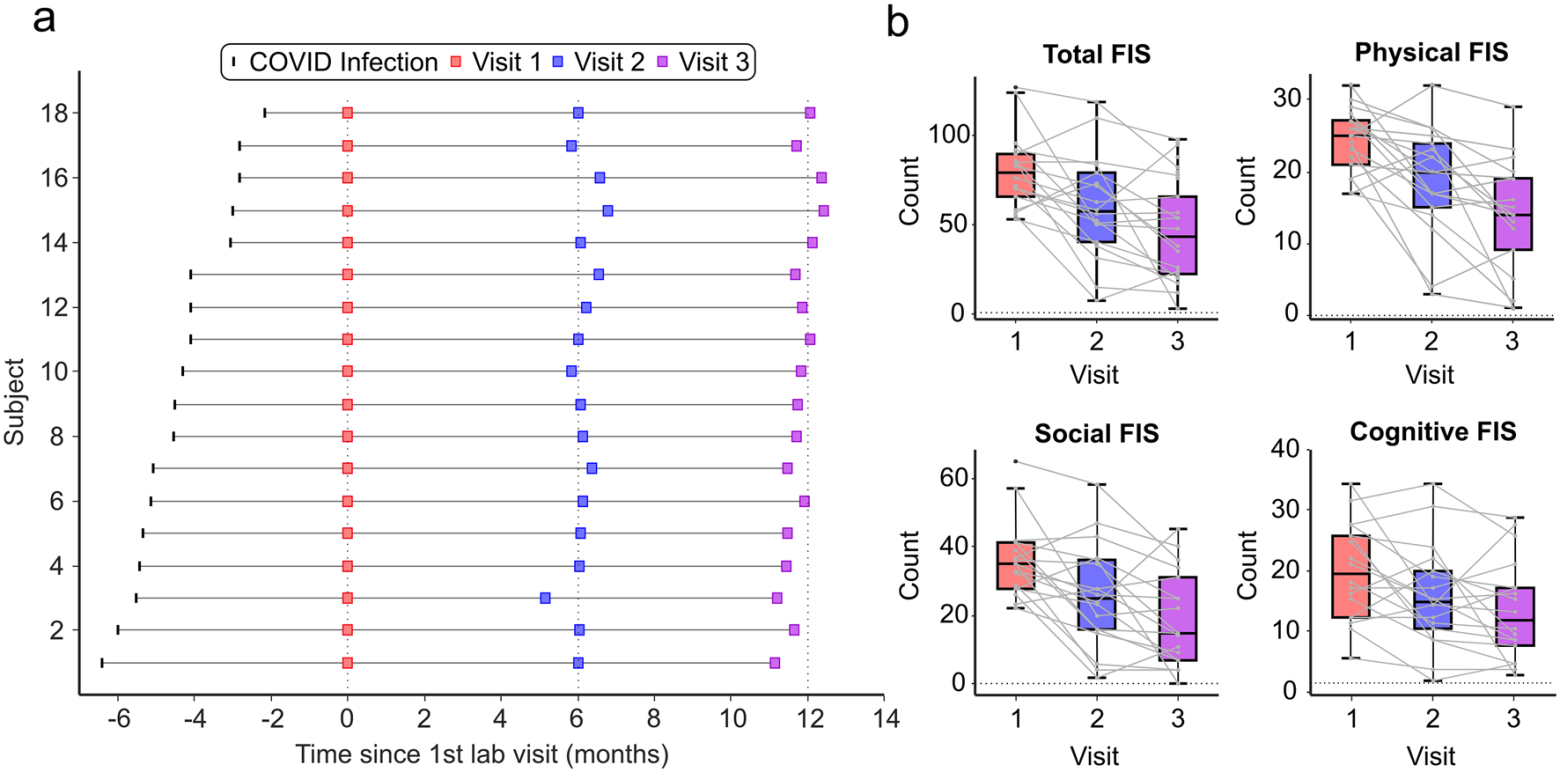
Timeline of visits and FIS scores. (**a**) Timeline of SARS-CoV-2 infection and subsequent visits to the lab for assessment for each participant. (**b**) Averaged FIS scores as box plots across all 18 participants for all visits; grey lines indicate individual subjects. Total FIS maximum count = 160; Physical = 40; Cognitive = 40; Social = 80.

Figure 1A shows the time line of the repeat visits of the 18 participants (13 females; age 48.1 ± 10.0 years) for whom we collected longitudinal data. Visits 2 (V2) and 3 (V3) were on average 6.02 ± 0.34 and 11.61 ± 0.36 months after visit 1 (V1).

### Fatigue impact scale

There was a significant (*p* < 0.0001, F = 13.3) decrease in the self-reported perception of the impact of fatigue across visits; mean FIS score declined from 80.3 at V1 to 60.2 at V2 and 46.1 at V3 (Fig. 1B). A similar trend was seen for all of the sub-domains of the FIS score: there was a significant average decrease over time in the cognitive FIS score (by 6.8 from V1 to V3, *p* = 0.009, F = 5.5), the social FIS score (decrease 16.9, *p* < 0.0001, F = 14.00) and the physical FIS score (decrease 10.5, *p* < 0.0001, F = 19.14). Overall, the majority (16/18) of participants had improved FIS scores between V1 and V3.

### Changes in biological measures

**Figure 2:**
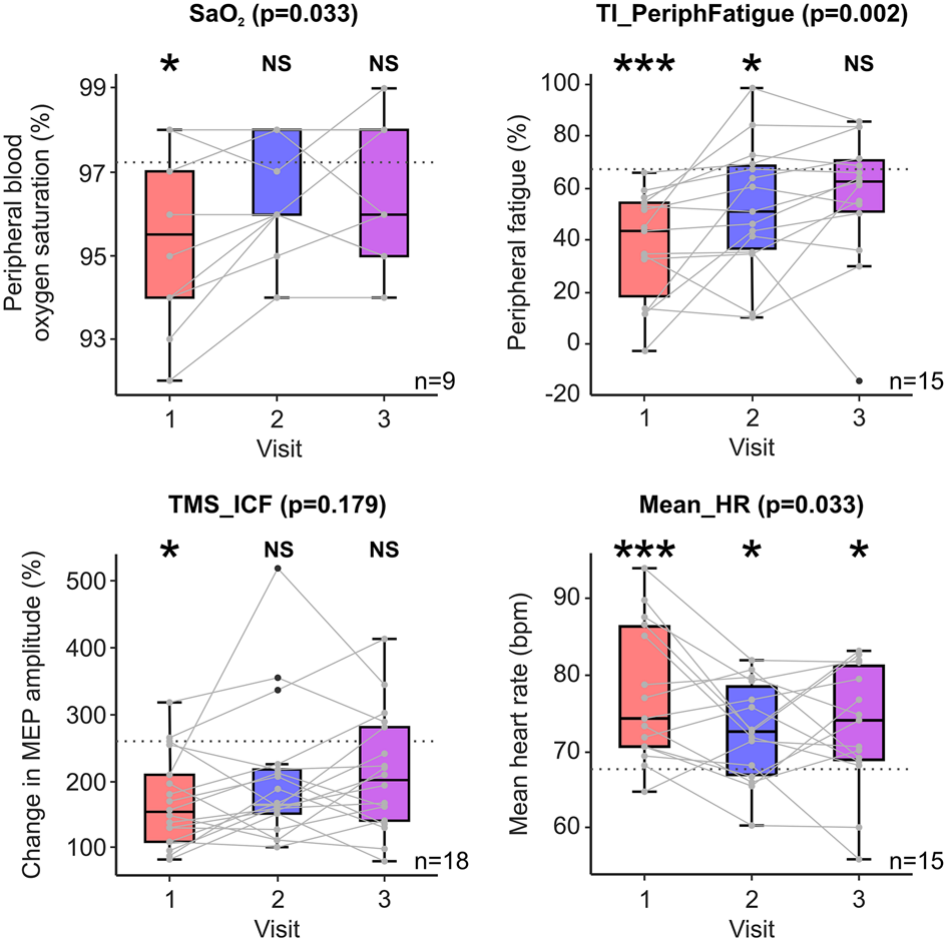
Changes in metrics over time. Box plot illustration of each metric (*SaO*_*2*_, *TI_PeriphFatigue*, *TMS_ICF*, *Mean_HR*) over time. The mean of the control cohort for each metric is illustrated by grey dotted lines. Bracketed P values above each plot are taken from repeated measure ANOVAs for each metric. Notations above each bar indicate significant (*P < 0.05; **P < 0.005; ***P < 0.0005) or non-significant (NS) difference between the post-COVID fatigue participants and controls (n = 51) for each visit.

Our earlier work showed that only a small number of measures (*TI_PeriphFatigue*, *TMS_ICF*, *Mean_HR*, *SaO*_*2*_) out of an extensive initial set were significantly different between controls and participants suffering from pCF. Only these measures were therefore repeated during V2 and V3 (Fig. 2). Over time there was a significant change in peripheral oxygen saturation (*SaO*_*2*_, *p* = 0.033, χ^2^(2) = 6.813), heart rate (*Mean_HR*, *p* = 0.033, F = 3.88), and peripheral fatigue (*TI_PeriphFatigue*, *p* = 0.002, χ^2^(2) = 12.4). In each case, values from people with pCF became more similar to controls (dotted lines in Fig. 2) with time. Using TMS to investigate the excitability of the primary motor cortex, we found that although intracortical facilitation became more similar to controls with each visit, this trend did not reach significance (*TMS_ICF*, p = 0.179, χ^2^(2) = 3.44).

Post hoc comparisons showed that *SaO*_*2*_ at V2 and V3 just misses becoming significantly increased from V1 (+ 0.71%, *p* = 0.047 and + 1.04%, *p* = 0.039 respectively, not significant after adjusting for multiple comparisons) and was not significantly different between V2 and V3. By contrast, *Mean_HR* at V2 and V3 significantly decreased relative to V1 (− 4.63 bpm, *p* = 0.024 and − 5.78 bpm, *p* = 0.035 respectively). *Mean_HR* was not significantly different between V2 and V3.

Peripheral fatigue (*TI_PeriphFatigue*) also showed significant improvements over time. At both V2 and V3, the metric *TI_PeriphFatigue* significantly increased (indicating less peripheral fatigue) compared to V1 (+ 17.49%, *p* = 0.007 and + 18.02%, *p* = 0.007 respectively), but values at V3 were not significantly different from those at V2.

Measuring peripheral fatigue required that subjects complete a sustained voluntary maximal contraction; this was terminated after 95 s, or earlier if the force exerted fell to below 60% of the starting level. Amongst controls, 47/51 subjects performed the full 95 s contraction, compared to 6/15 people with pCF at V1 and 15/15 at V2 and V3; the proportion was significantly different from controls only at V1 (χ^2^(1,N = 66) = 19.9, *p* < 0.0001). The maximum voluntary contraction (measured as the best of three short contractions before the sustained contraction) was 195.1 ± 77.1 N for controls, 173.1 ± 59.2 N at V1, 203.2 ± 68.2 N at V2 and 183.7 ± 87.4 N at V3 for people with pCF. However, the change over time was not significant. The duration of the sustained contraction was 93.2 ± 10.0 s for controls, 70.9 ± 24.1 s at V1, 95.3 ± 0.5 s at V2 and 95.4 ± 0.7 s at V3 for the pCF cohort. This increase over time for pCF was significant (*p* = 0.031, χ^2^(2) = 6.962). The magnitude of decline in contraction force (measured as the maximal force generated at the beginning of the sustained contraction subtracted from the force generated at the end) was − 46.3 ± 47.4 N for controls, − 40.4 ± 29.5 N at V1, − 44.8 ± 35.5 N at V2 and − 33.5 ± 33.7 N at V3 for the pCF cohort. This change over time was not significantly different.

Although both FIS scores and the biological metrics improved significantly over the course of the 12 month study, surprisingly there was no significant correlation between change in FIS score and changes in any of these measures (r^2^ values: *TMS_ICF* 0.00063, *SaO*_*2*_ 0.011, *Mean_HR* 0.17, *TI_PeriphFatigue* 0.086, all *p*-values > 0.05). A similar lack of correlation was found with each of the FIS sub-scores whose changes are illustrated in Fig. 1b.

As stated previously, the four metrics repeated during V2 and V3 were significantly different from controls at V1 (*TMS_ICF* *p* = 0.006, *SaO*_*2*_ *p* = 0.008, *Mean_HR* *p* < 0.001, *TI_PeriphFatigue* *p* < 0.001). At both V2 and V3, only *Mean_HR* remained significantly different from controls (V2 *p* = 0.046, V3 *p* = 0.035), whilst *TI_PeriphFatigue* was only significantly different from controls at V2 (*p* = 0.021), but not V3*. TMS_ICF* and *SaO*_*2*_ were not significantly different from controls. Furthermore, the maximum voluntary contraction was not significantly different between people with pCF and controls for any of the three visits.

### Additional measures of muscle physiology

**Figure 3:**
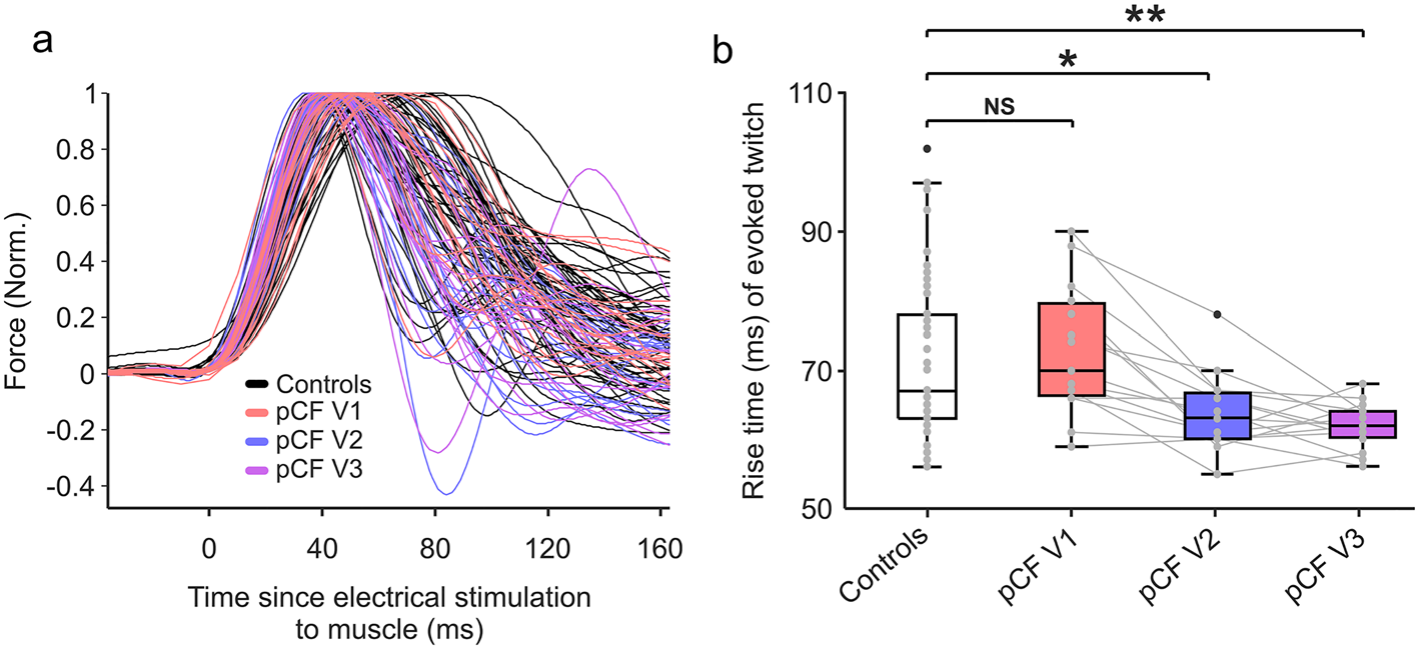
Rise Time. (**a**) Raw traces of twitch responses to direct electrical stimulation of biceps for each participant, for all cohorts. The trace for each participant was normalised to the maximal force generated by each individual. *RiseTime* is calculated as time from electrical stimulation to maximal force generated. (**b**) Box plots showing *RiseTime* data for controls (n = 51) and post-COVID fatigue participants (n = 15) for each visit. NS, not significant; *P < 0.05; ** *P* < 0.005.

To investigate the potential mechanisms underlying improvements seen with peripheral fatigue over time, we made one further measure of muscle physiology—the rise time of a maximal twitch (*RiseTime*). This refers to the time taken from direct muscle stimulation to the peak force generated (measured from the biceps, at rest at the start of the twitch interpolation protocol; Fig. 3A). Although, relative to controls, there was no significant difference at V1 (*p* = 0.410), *RiseTime* was significantly reduced compared to controls at both V2 (*p* = 0.010) and V3 (*p* < 0.001). Furthermore, *RiseTime* of the pCF cohort significantly fell over time (*p* = 0.001, χ^2^(2) = 13.661). Post hoc comparisons showed that at both V2 and V3, *RiseTime* significantly decreased relative to V1 (− 9.8 ms, *p* < 0.001 and -11.8 ms, *p* < 0.001 respectively). *RiseTime* was not significantly different between V2 and V3.

## Discussion

A substantial proportion of people in the UK (2.9%) continue to suffer from longer-term sequalae of SARS-COV-2 infection (Long COVID)^16^. Of various lingering symptoms such as difficulty concentrating (51%), muscle aches (49%) and shortness of breath (48%), persistent fatigue is one of the most common (72%)^16^. Persistent fatigue after a (non-SARS-CoV-2) viral infection is well known to the clinician but is also a hallmark of several autoimmune and neurological disorders, suggesting a link with nervous system dysfunction. Recent work has shown that even a mild to moderate COVID-19 infection can cause dysregulation in the nervous system^17^.

In our previous study^12^, we provided evidence that measures of the central (intracortical facilitation, *TMS_ICF*), peripheral (peripheral fatigue, *TI_PeriphFatigue*) and autonomic (mean heart rate, *Mean_HR* and peripheral oxygen saturation, *SaO*_*2*_) nervous systems were different between people with pCF compared to sex- and age-matched controls. However, from these findings one cannot infer causation; measures could have been different prior to the infection (and thus conferred an increased likelihood of developing pCF) or the abnormal measures could be a consequence of the infection (and hence might be useful as a biomarker for tracking changes in fatigue). To address this, we repeated measurements 6 months and 12 months later. As a group the reported impact of fatigue decreased, and our metrics returned to or were returning to normal. This suggests that the changes were mediated by SARS-CoV-2 infection, rather than being a pre-existing long-term trait amongst the pCF sufferers.

The most significant change over time was observed in muscle fatigue. The metric *TI_PeriphFatigue* increased (signifying less peripheral fatigue) from 37% at the first lab visit to 55% one year later, indicating that muscles undergo significant physiological changes during recovery. Acute SARS-COV-2 infection can affect skeletal muscle through several mechanisms. Firstly, the virus can cause direct muscle cell damage by entering host cells via the ACE-2 receptor and TMPRRSS2 protein^18^, both of which are expressed by musculoskeletal tissue^19^. In muscle, there is some evidence that SARS-CoV-2 may impair mitochondrial function directly causing critical illness myopathy^20^. Mitochondrial impairment has recently been shown to occur in long COVID, even when patients had not been hospitalised^9,10^. Secondly, patients can develop acute respiratory distress syndrome^21,22^, characterised by severe hypoxaemia, thereby diminishing systemic oxygen supply to muscle tissue. Hypoxia significantly affects mitochondrial activity, compromising muscle energy generation needed for protein synthesis and muscle contraction^23,24^. Thirdly, SARS-COV-2 infection can cause a hyper-inflammatory state in muscle^25,26^. Physiological muscle stimulation during physical activity leads to the production of myokines, which normally induce an anti-inflammatory environment. However, in the presence of the SARS-CoV-2 virus, myokine production instead stimulates a prolonged muscular inflammatory environment^25,26^. Inflammation, similar to hypoxia, also impairs mitochondrial function^27^ and reduces protein synthesis, and thus has the potential to induce long-term sarcopenia^9,28^.

Aside from the potential direct actions of SARS-CoV-2 on mitochondria (see above), there may also be indirect effects of SARS-COV-2 on mitochondrial function that contribute to ongoing fatigue encountered in long COVID. For example, there is evidence in critical illness that mitochondrial haplotype conveys survival advantage^29^. In sepsis, mitochondrial dysfunction results in diaphragmatic myopathy^30^, and the extent of mitochondrial DNA depletion, measurable in mononuclear cells, correlates with the severity of critical illness^31^ It is thus possible that those who develop pCF might have subclinical mitochondrial dysfunction, subsequently unmasked by SARS-COV-2 infection, leaving them with systemically impaired mitochondrial function, as is typical of the pattern in mitochondrial disease^32^, potentially explaining the broad spectrum of symptoms experienced by patients suffering from long COVID.

There is clear evidence for a specific role of mitochondrial dysfunction both in critical illness myopathy^33,34^, and in long COVID^9,10^. Metabolic adaptations include a shift away from energy generation in mitochondria through oxidative phosphorylation and towards anaerobic glycolysis, where pyruvate is converted into lactate^9,35^, and phosphocreatine breakdown; these changes in turn lead to the accumulation of metabolites that promote inflammatory processes^36^ and the generation of muscle fatigue^37^.

Our cohort with pCF showed significantly less peripheral fatigue 6 months and 12 months after they were first assessed. Interestingly, as the pCF cohort recovered from the symptoms of fatigue, their twitch tension rise times became faster than controls (Fig. 3B). This suggests that the improvement over time in muscle fatigability (corresponding to an improved ability of muscle to generate force with continued contraction) might be due partly to a process of adaptation rather than a complete return to baseline physiology*.* One recent report showed an increase in the proportion of type II muscle fibres in long COVID^9^, although another failed to find a significant difference in fibre type proportions^10^. A similar change in fibre type is recognized in mitochondrial myopathy, where histopathology is reported to show an increased ratio of type II to type I fibres^38^. More recently, proteins involved in mitochondrial fusion/fission have been implicated in the intracellular signalling processes regulating fibre type switching^39^, as observed in response to exercise^40^. Type II fibres have high fatigability, because of their higher reliance on anaerobic rather than aerobic metabolism, and faster single fibre twitch times because of a higher rate of cross-bridge cycling and associated ATP breakdown^41^. A shift towards type II fibres could therefore lead to the shorter muscle twitch time seen here. However, it must be noted that the twitch time was not different between controls and long COVID in the first visit of our study; the difference only developed with time as pCF recovered. A shift in fibre type cannot therefore be the primary underlying cause of pCF.

Our longitudinal data may explain the difference in recent published findings on fibre type composition. In the publication^10^ which found no difference in fibre type proportions between controls and long COVID, patients were recruited on average 8 months after their acute infection, corresponding to a time point mid-way between V1 and V2 in the present study. By contrast, the paper^9^ which reported fibre type differences recruited a mean of 16.6 months after acute infection, close to our V3. Our results are therefore in close agreement with this literature. We do not know whether rise time (or by inference fibre type composition) would eventually return to baseline values over a time course of more than the one year interval of our study.

Changes in peripheral fatigue had a functional impact. Whereas almost all (92%) of controls were able to hold a sustained contraction above 60% of original maximal force for 95 s, only 40% of people with pCF could do this at V1, but all managed it at V2 and V3.

As well as changes in peripheral fatigue, other measures also returned closer to control values. Blood oxygen saturation (*SaO*_*2*_) rose; this is likely to reflect a slow improvement in cardio-pulmonary function in the aftermath of the acute COVID infection. A recent study^10^ found no difference between controls and long COVID sufferers in measures of lung function. However, it should be emphasised that the difference we observed in *SaO*_*2*_ at V1 was small – a mean of 95.3% in patients versus 97.2% in controls^12^; such small differences could easily be obscured in more nuanced measures. The resting heart rate was elevated at V1 but reduced to be closer to control levels by V3; this suggests that the balance between sympathetic and parasympathetic drive returned closer to normal as patients recovered. Finally, although the changes in intracortical facilitation over time failed to reach significance, there was clearly a trend back towards control values. In all cases our findings were consistent with a progressive normalisation of autonomic, peripheral and central nervous system metrics. However, our result with peripheral fatigue and twitch time should provide caution; we cannot know whether changes in blood oxygen saturation, heart rate or cortical facilitation reflect a normalisation of the underlying physiology, or the results of secondary compensation whereby the primary pathology is overlain with a homeostatic response.

Although our biological metrics returned to levels that were compatible with controls and reported impact of fatigue reduced (Fig. 1B), we found no significant correlation between changes in these two. This is perhaps not surprising. How an individual copes with fatigue—and thus assesses its impact on their life—will probably be affected by psychological factors such as level of resilience or social considerations, such as varied access to support systems. The magnitude of change in FIS score, measured by a subjective questionnaire, is likely to be influenced at least as much by these factors as the underlying pathology, which may explain the lack of correlation with more objective metrics.

Our findings have significant, but indirect, clinical implications. Understanding the trajectory of neurophysiological recovery in long COVID has the potential to inform personalized treatment approaches and rehabilitation strategies. In particular, the measurement of peripheral fatigue is simple and can be achieved with low-cost equipment. This could deliver an objective assessment, to be interpreted alongside subjective measures such as the FIS score.

### Supplementary Information

Supplementary Legends.

Supplementary Information 1.

Supplementary Information 2.

## Data Availability

A spreadsheet containing values for all subject measurements across visits is available in Supplementary Material.
