# Supplementary material for: Recovery of neurophysiological measures in post-COVID fatigue: a 12-month longitudinal follow-up study
Source: Sci Rep. 2024 Apr 17;14:8874. doi: 10.1038/s41598-024-59232-y (PMC11024107; doi:10.1038/s41598-024-59232-y)
Supplement: Supplementary file 1 — Supplementary Legends. [file 41598_2024_59232_MOESM1_ESM.docx]

**Recovery of Neurophysiological Measures in Post-COVID Fatigue – a One Year Longitudinal Study**

**Natalie J. Maffitt*, Maria Germann, Anne M.E. Baker, Mark R. Baker,**

**Stuart N. Baker and Demetris S. Soteropoulos**

Faculty of Medical Sciences, Newcastle University, UK

* Corresponding author: n.maffitt@newcastle.ac.uk

**Supplementary Tables**

**Supplementary Table 1. Participant information and values of each measure sampled in this study, for both control and pCF cohorts across all visits.** This table shows the biometric data, FIS scores, and neurophysiological measurements of each individual participant. Each sheet of the spreadsheet corresponds to the control, pCF visit 1, pCF visit 2, and pCF visit 3 cohorts.

**Supplementary Table 2. Statistical test values for each measure sampled in this study for each cohort and visit.** This table shows the summary values for each measure and cohort separately. The mean, standard deviation and standard error of the mean are shown alongside the total number of participants per cohort.
